# Case Report: Abomasum Impaction in Beef Cattle Due to High Intake of Distillers' Grain and Treatment Options in Southwest China

**DOI:** 10.3389/fvets.2021.615871

**Published:** 2021-05-24

**Authors:** Kang Yong, Zhengzhong Luo, Qinwen Yang, Chuanshi Zhang, Qianlan Zhou, Hua Jiang, Yong Zhang, Suizhong Cao

**Affiliations:** ^1^College of Veterinary Medicine, Gansu Agricultural University, Lanzhou, China; ^2^College of Animal Science and Technology, Chongqing Three Gorges Vocational College, Chongqing, China; ^3^The Key Laboratory of Animal Disease and Human Health of Sichuan Province, College of Veterinary Medicine, Sichuan Agricultural University, Chengdu, China; ^4^Zunyi Beef Industry Engineering Technology Research Center, Zunyi, China

**Keywords:** abomasum impaction, cattle diseases, distiller's grains, diagnosis, treatment

## Abstract

In Southern China, distillers' grain is the main feed ingredient for small beef cattle farms. High intake of distillers' grain may lead to abomasum impaction, a disorder caused by the accumulation of solid content within the organ. For treatment, there are non-surgical and surgical options. In this study, we aimed to describe the clinical presentation, diagnosis, and treatment of beef cattle with abomasum impaction due to high intake of distillers' grain. Forty-nine Simmental beef cattle from 13 farms in Chongqing, China, were diagnosed with abomasum impaction. Animals were male, aged ≤2 years, and weighed between 200 and 350 kg. In this retrospective study, information on distillers' grain intake and clinical data were collected for 49 beef cattle diagnosed with abomasum impaction. The animals were treated between 2011 and 2019 with either non-surgical therapy or surgery. Animals diagnosed with mild abomasum impaction (*n* = 14) fully recovered after non-surgical treatment. Among moderate cases (*n* = 19), 12 cattle recovered after non-surgical treatment (63%), while the remaining seven did not respond well and underwent surgery. Three of those animals were subsequently cured (3/7). Among the severe cases (*n* = 16), four cattle were cured after non-surgical treatment (25%) (4/16). Of the remaining 12 cattle, six were slaughtered, and six died after surgery. Non-surgical treatment is efficient for mild abomasum impaction caused by a high intake of distillers' grain and may be considered for both moderate and severe cases. However, the treatment success rate for more severe cases decreases as the disease severity worsens.

## Introduction

Distillers' grain is an important feed ingredient for intensive beef farming in Southern China because of high yield, low prices, and high net weight gain (1, 2). However, this grain is not ideal in large quantities, and to avoid potential health issues, the amount of distillers' grains should be adjusted according to the requirements of cattle (3). In the diet for beef cattle weighing about ~400 kg, the amount of wet distillers' grains added is generally controlled below 10 kg per cow per day (4). Unfortunately, in the actual breeding process, this rule is not strictly followed, and distiller's grain is often added in excess.

Abomasum impaction (AI) is a disorder where the organ becomes enlarged because of accumulation of solid, dry matter (5–8). AI can cause dehydration, disturbance of electrolyte balance, alkalosis, and progressive weight loss (5, 9). The etiology of this disorder includes traumatic reticuloperitonitis, vagal indigestion, adhesion of the abomasum with the rumen or the ventral part of the abdominal wall, and ingestion of non-food items (5, 7). According to the nature of the obstruction, AI is divided into food obstruction and foreign body obstruction (5, 9). AI may develop in beef cows when cattle have decreased water intake during the winter and are fed poor-quality roughage (10, 11), including a high quantity of distillers' grain. Studies have shown that the consumption of distillers' grain in cattle can cause increased production of volatile fatty acids in the rumen (12). After a large amount of volatile fatty acids enter the abomasum through the rumen, it causes weaking of smooth-muscle contractions (13), which may lead to AI. Recent studies have noted that phytobezoars, gravel, wool balls, placenta, plastic, string, almond husk, and pineapple pulp can cause AI in cattle (5, 7, 9, 11, 14). However, there have been no reports of distillers' grain causing AI in beef cattle.

In this study, the diagnosis and treatment of 49 cases of AI in beef cattle, possibly caused by high intake of distillers' grain, were analyzed in Southwest China. The results presented here may provide a reference for the prevention and treatment of this disease.

## Materials and Methods

### Selection of Abomasum Impaction Cases

Forty-nine cases of AI in beef cattle were selected for this study. All animals originated from Chongqing and were submitted to examination by experienced veterinarians between 2011 and 2019. All animals were male and aged ≤2 years and had an average body weight of 200–350 kg. All animals were subjected to a high intake of distillers' grain as a normal part of their fattening feed. The animals were fed twice a day, at ~8 am and 5 pm. All diseased beef cattle had consumed a total of more than 15 kg of distillers' grains daily for more than 1 week. The animals were in the early stages of fattening. Cattle were fed in their respective enclosures and allowed limited exercise. No cattle were treated until they had been diagnosed with AI. All cattle were diagnosed with abomasum impaction in the field and treated in the College of Chongqing Three Gorges Vocational Veterinary Teaching Hospital.

The main clinical symptoms in the early stage (1–4 days) of the disease include reduced feed intake and reduced rumination, reduced auscultation of rumen and omasum motility, palpation of the abomasum shows slight enlargement but it remains soft, no significant abnormality in the abdominal circumference, dry feces, similar to abacus beads, and increased water consumption. The main clinical symptoms of the disease in the middle stage (5–10 days) include nose dryness, decreased appetite and increased water consumption, substantial enlargement of the abdominal circumference, the rumen appears full with content or contains a large amount of fluid (impact palpation, water vibrations are present), rumen and omasum sounds are not present, and intestinal sounds are weak; the urine output is limited, and during defecation only a small amount of mushy feces is discharged. The clinical symptoms at the final stage of the disease (longer than 10 days) include depression, inverted hair, and sunken eyes; loss of appetite and rumination and cracked nose, the right mid-abdomen to the lower back shows limited swelling. The diseased cow shows dodging behavior, and at the same time, the body of the abdomen is markedly expanded and hard. The animal cannot defecate or can only discharge a small amount of tan foul-smelling feces, mixed with mucus or purple-black blood and blood clots. The urine volume is limited and the urine concentrated, yellow or dark yellow, with a strong odor. Some of the diseased animals cannot stand. According to the clinical symptoms, cattle were divided into mild, moderate, and severe AI (Table 1). For the observation of pathological changes associated with AI, the organs were observed when the symptomatic cows that did not respond to treatment were slaughtered.

**Table 1:** Clinical data from 49 AI cases in beef cattle.

**Clinical signs**	**Case stage**		
	**Mild (*n =* 14)**	**Moderate (*n =* 19)**	**Severe (*n =* 16)**
Reduced feed intake	+	++/+++	++++
Reduced rumination	+	++/+++	++++
Abomasum expansion	+/++	+++/++++	++++
Abomasum hardness	−/+	++/+++	++++
Feces reduction	−/+	++/+++	+++/++++
Decreased urination	−	+/++	++/+++
Abdominal distension	−/+	++/+++	++++
Rumen fluid	−	+/++	+++/++++
Decreased rumen motility	+/++	++/+++	+++/++++
Drinking more water	−/+	++/+++	+++/++++
Dehydration	−	+/++	+++/++++
Depression	−	+/++	+++/++++

–*, Negative clinical findings; +, Positive clinical findings; the number of “+” symbols indicates degree of the finding*.

### Clinical Examination

Thorough physical examinations of diseased beef cattle were carried out according to the standard methods described by Cockcroft et al. (15), including body temperature, heart rate, respiratory rate, rumen movement, and defecation.

### Treatment of AI

**Figure 1:**
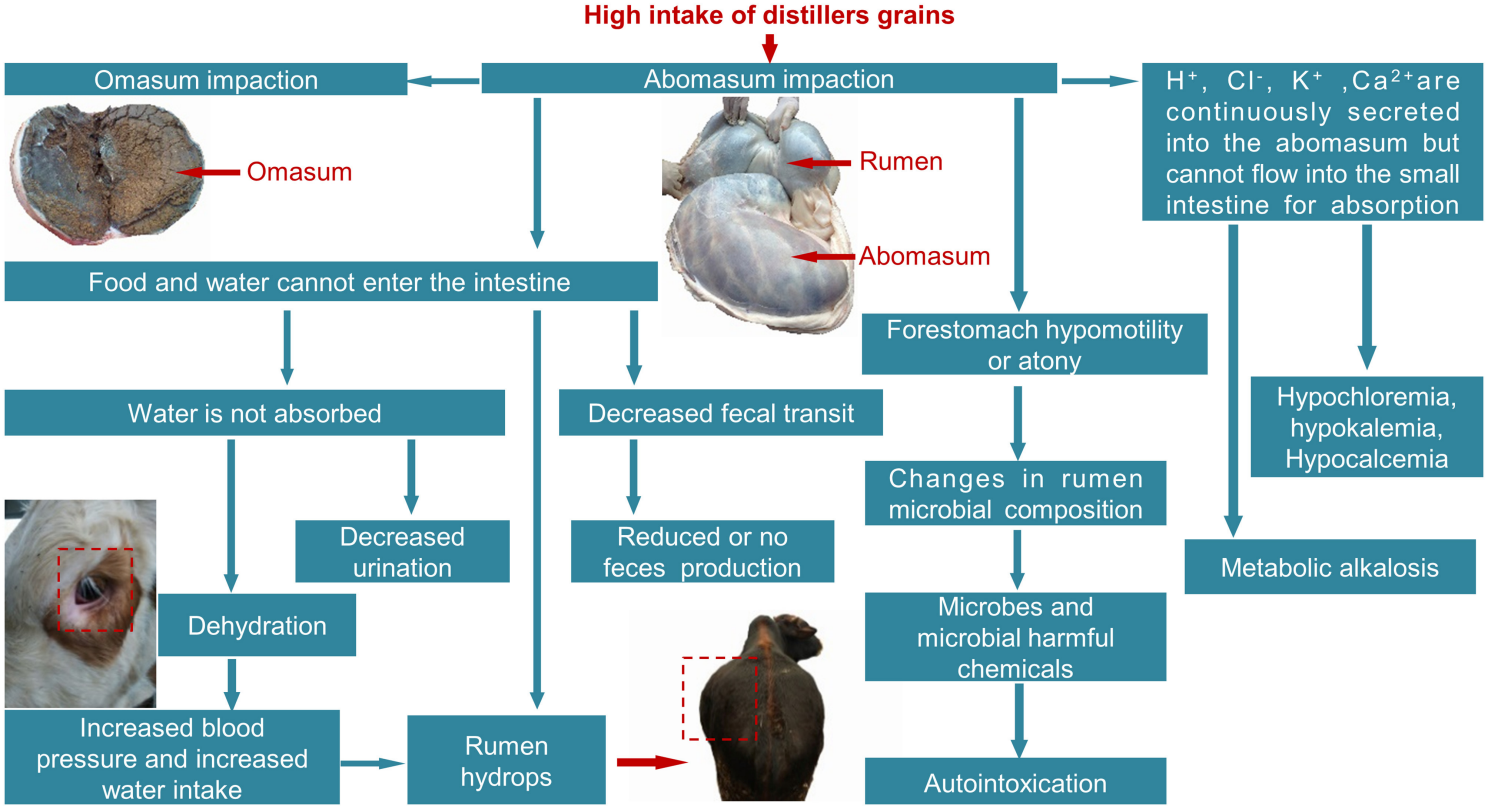
Clinical features and metabolic alterations in AI. Rumen, abomasum, and omasum are indicated with red arrows. The abomasum volume is 2–3 times larger than normal, and omasum contents are hard with necrotic leaflets. Omasum impaction is secondary to ruminal impaction.

Considering the cost of treatment and requests from the cattle owner, all cattle received non-surgical treatment first. Non-surgical treatment involves oral or intramuscular or intragastric injection of drugs that promote gastrointestinal contraction to facilitate the discharge of the abomasum contents. Intravenous rehydration is administered to beef cattle who are dehydrated and have metabolic alkalosis and hypokalemia. Surgical treatment was performed only when non-surgical treatment failed. Surgeries involved abomasotomy or rumenotomy with flushing of the abomasum and were carried out in compliance with China's Animal Welfare Protection Law. Treatment options were selected according to clinical features and metabolic alterations in AI (Figure 1). Specific treatment measures are shown in Table 2. For the purposes of this study, a successful treatment outcome implied that the obstruction was cleared, including relief of pyloric spasms and the discharge of the abomasum contents.

**Table 2:** Treatment protocols for 49 beef cattle.

**Case stage**	**Treatment protocols**
Mild (*n =* 14)	Rhubarb soda tablets (0.3 g)^a^ 250–500 tablets, vegetable oil 1,000–3,000 mL, irrigate after mixing, 1 time/day; 10–20 g of vitamin C and 10–20 mg of neostigmine methylsulfate injection were given intramuscularly, 2 times/day. Continuous treatment until a large amount of loose stools mixed with distilled grain are discharged.
Moderate cases (*n =* 19)	In the first step, after the rumen liquid and gas are led out through a gastric tube, 250–500 pieces of rhubarb soda tablets (0.3 g) and 1,000–3,000 mL of vegetable oil are injected once a day. In the second step, 500–1,000 mL of 25% sodium sulfate solution and 500–1,000 mL of vegetable oil were mixed and injected intragastrically once per day. After injection, the abomasum area was massaged, and the cattle were forced to exercise. Third, injections including: (1) 1,500–2,000 mL of 10% glucose injection, 200–500 mL of 10% calcium gluconate, 10–20 g vitamin C; (2) 10% concentrated chlorine 300–500 mL, 5–10 g of 10% potassium chloride; (3) 2,000–3,000 mL Ringer's solution. (1–3) one-time intravenous injections; (4) 10–20 mg of neostigmine methylsulfate injection, one intramuscular injection, 2 times/day. If the above three steps are treated for 2 days, and the cattle are still unable to excrete feces, surgical treatment is immediately performed (step 4). If bowel movement is recovered (feces discharged) after 2 days of treatment, continue to treat with this method for 3–5 days until the cow is cured.
Severe cases (*n =* 16)	The treatment steps are the same as those in the moderate cases. For cows that cannot stand and have significant pain, intramuscular injection of meloxicam at 0.5 mg/kg

a *Rhubarb soda tablets (0.3 g) contain 0.15 g of rhubarb, 0.15 g of sodium bicarbonate, and 0.001 mL of peppermint oil*.

### Statistical Analyses

Data were analyzed using SAS (version 9.4; SAS Institute Inc., Cary, NC). Mean differences in body temperature, heart rate, respiratory rate, and serum biochemical analysis were calculated using unpaired two-tailed Student's *t*-test. Data are presented as means and standard errors. Differences were considered statistically significant when *P* < *0.05* or *P* < *0.01*.

## Results

### Diagnosis of AI Cattle

A total cure was obtained for 67% of the cases. After non-surgical treatment, 30 cattle fully recovered (61%). Thirteen cattle were submitted to a surgical procedure, but only three cattle recovered (23%). The specific treatment outcomes are shown in Table 3.

**Table 3:** Treatment results of 49 beef cattle.

**Case classification**	**Treatment results**
Minor cases (*n =* 14)	All 14 beef cattle recovered
Moderate cases (*n =* 19)	After the first three steps of treatment, 12 cattle were cured, and another 7 cattle were submitted to surgery. Four of the cattle were treated with abomasotomy, one was cured, and three were eliminated because of gastroenteritis. Another three cattle were treated with rumenotomy with flushing of the abomasum. Two cattle recovered and one died.
Severe cases (*n =* 16)	Four beef cattle were cured using the first three steps. After the first three steps of 12 beef cattle failed, six cattle were slaughtered, and the remaining six died after choosing the fourth step (four beef cattle were treated with abomasotomy, and two beef cattle were treated with rumenotomy with flushing of the abomasum)

### Examination of AI Cattle

The body temperature, respiratory rate, heart rate, and serum biochemical analysis of all the cattle were within the normal range, and there were no statistically significant differences among the mild, moderate, and severe cases. Common clinical symptoms included reduced rumen peristalsis and reduced feed intake. The mean body temperature was 38.65 ± 0.68°C [reference range, 37.5–39.5°C], the mean heart rate was 78.60 ± 8.51 beats/min [reference range, 60–80 beats/min], and the mean respiratory rate was 25.79 ± 1.83 beats/min [reference range, 10–30 beats/min]. Twelve cows had heart rates faster than 100 beats/min, and seven cows had respiratory rates over 30 beats/min. Thirty-one cattle were dehydrated (i.e., recession of the eyes in the orbits and skin tent duration >2 s). The mean serum calcium concentration was 2.47 ± 1.58 mmol/L [reference range, 2.35–3.05 mmol/L], mean serum potassium concentration was 4.23 ± 2.17 mmol/L [reference range, 3.9–5.8 mmol/L], and mean serum chloride concentration was 99.58 ± 3.53 mmol/L [reference range, 97 to 111 mmol/L].

There were 28, 23, and 16 beef cattle with hypochloremia, hypokalemia, and hypocalcemia, respectively. Notably, in 11 cows, the total bilirubin increased more than 12 mmol/L [reference range, 0–12 mmol/L] and in eight cows, cholesterol increased more than 5.16 mmol/L [reference range, 1.16–5.16 mmol/L].

After a post-mortem examination of the sick cows, it was found that the abomasum was extremely dilated, and the volume increased remarkably, up to 2–3 times larger than normal (Figure 1). The abomasum was blocked because of dry content. In the ischemic area, the abomasum wall was thin and easy to tear. Abdominal mucosal inflammation, infiltration, necrosis, and shedding were also observed. In some cases, scattered bleeding spots or ulcers were detected at the pyloric area and bottom of the abomasum. The size of the omasum was increased, its contents were hard, the leaflets were necrotic, and the mucosa was shedding around extensive areas (Figure 1).

## Discussion

### Diagnosis of Abomasum Impaction

Initially diagnosing AI is difficult as it is easily confused with diseases such as forestomach atony, rumen impaction, rumen effusion, omasum impaction, large bowel constipation, and peritonitis. Differential diagnoses should consider the following points: long-term or excessive consumption of distillers' grain, insufficient addition of green fodder or hay to the feed, and lack of exercise. Additionally, upon auscultation of the rumen and omasum, one can observe that peristaltic sounds disappear. Furthermore, upon auscultation of the left palate with simultaneous percussion of the first to the fifth rib on the left or the first to the second rib on the right leads to steel pipe sounds (11, 14). The rumen also appears full or contains a large amount of fluid (impact palpation with sloshing sounds), and the abomasum area is sensitive and hard. In severe cases, the abomasum area shows swelling, and feces and urine cannot be excreted, with large doses of laxatives having no effect. Cattle are severely dehydrated, the eyeballs are sunken, the nose is dry, and the abdominal circumference is enlarged. Some cattle cannot stand (16). According to the early clinical symptoms (Table 1), timely diagnosis and treatment can improve the cure rate and save the cost of treatment. When cattle show clinical symptoms such as loss of appetite, repeated ruminal tympany, and constipation, especially following long-term feeding with high doses of distillers' grain, the veterinarian should suspect AI and, if necessary, perform abdominal puncture or right abdominal examination (17). In this study, 12 cows with mild disease were all diagnosed in time, so they all recovered after treatment with the lowest cost of treatment.

### Ancillary Tests of Abomasum Impaction

The prognosis of sick cattle should be determined based on the clinical diagnosis results to avoid unnecessarily increasing the treatment costs. Biochemical tests were performed on 20 buffalo with AI, and it was found that levels of Cl^−^, K^+^, malondialdehyde, superoxide dismutase, and uric acid were significantly lower in the blood of calves who did not survive than in the blood of those who survived (9). In the same study, the authors reported that total protein, albumin, creatinine, urea, aspartate aminotransferase, glutamine transferase, and total bilirubin levels were higher in the blood of calves who did not survive than in the blood of surviving calves (9). The results of that study may provide additional insight into the diagnosis and prognosis of sick cattle. In order to make the prognostic evaluation more convincing, the next step is to collect a large number of cases, monitor biochemical indicators, and combine clinical symptoms to establish a comprehensive evaluation mechanism.

### Treatment Methods of AI

For the non-surgical treatment of AI reported in this study, oral administration of oil laxatives was preferred instead of salt laxatives. This is because salt laxatives increase rumen osmotic pressure and the consequent risk of dehydration. While using large-dose laxatives, the timely administration of intravenous fluids is important to prevent dehydration. Additionally, acid–base balance (metabolic alkalosis) and electrolyte balance (hypokalemia, hypochloremia, hypocalcemia) are also critical (5, 18). The solutions used with this purpose are mainly Ringer's solution, potassium chloride, and calcium gluconate. In mild cases, when there was still some appetite and dry stools were excreted with no severe dehydration, the abomasum was not completely blocked; therefore, no infusion treatment was used. In these cases, just the oil laxatives, rhubarb soda tablets, and neostigmine methylsulfate sufficed to promote gastrointestinal motility, and the sick cows were cured. Intraluminal injections of laxatives, saline, and Chinese medicine to treat AI have been reported (19, 20). After injecting laxatives to 35 cows, only 16 cows were unblocked. It is difficult to evaluate whether comprehensive therapy works or whether simple abomasum injection works. Furthermore, studies have reported that oxidative stress occurs in AI (9). Vitamin C as a non-enzyme antioxidant can directly scavenge free radicals, which can relieve cell damage caused by oxidative stress (21). Therefore, vitamin C injection has been previously used during treatment. AI has been surgically treated by means of abomasotomy (22), rumenotomy with flushing of the abomasum (23), laparotomy, and massage (5, 24) pyloroplasty or pyloromyotomy (25), and anastomosis between the pyloric antrum and duodenum (26). One study performed right flank laparotomy and abomasal massage in 73 cattle (5). After surgery, 54 (74%) cattle received 3–4 L of mineral oil, postoperatively, daily for 1 to 5 days. With this strategy, short-term survival rate was significantly higher for cows with impaction of the pyloric antrum alone (42/45 [93%]) than for cows with impaction of the body and antrum (12/24 [50%]). The results of this study suggest that laparotomy with massage of the impacted pyloric antrum in combination with postoperative administration of mineral oil by ruminal intubation is an effective treatment. This surgical method has not been reported in China. The authors believe that this method is feasible because it does not directly damage the abomasum and is beneficial to avoid postoperative complications such as abomasitis and abomasum ulcers.

### Current Status of AI Therapy

At present, China mainly adopts the first two types of surgical methods, abomasotomy and rumenotomy with flushing of the abomasum. These two surgical methods have their own advantages and should be selected according to the size of the beef cattle and the severity of AI (27, 28). The rumen incision method demands a simple operation. It is suitable for small-sized cattle with no severe obstructions that can stand. In traumatic reticuloperitonitis, this procedure can solve the primary disease. For cattle that are large, cannot stand, or have severe obstruction, they should be treated decisively with abomasotomy. Some studies have successively reported that the two methods mentioned above are reliable in the treatment of AI (27–30). The authors believe that if non-surgical treatment is ineffective, those with moderate cases should be promptly submitted to the second surgical method; for severe cases, surgery should be abandoned (six cattle died after surgery) to reduce treatment costs. We believe that it is important to evaluate if immediate surgical treatment could improve the cure rate once severe cases are diagnosed. This would clarify whether the high rate of surgery failure in this study was attributable to the initial use of non-surgical therapies, which may have aggravated the disease and delayed the optimal treatment.

Finally, an adequate ratio of distillers' grain in cattle feed is a fundamental measure to prevent AI. Decreasing the amount of distillers' grain can prevent the occurrence of AI in beef cattle. The producers should also evaluate the quality of the distillers' grain, inspecting the feed for the presence of mud or mildew. When using distillers' grain, an appropriate amount of grass or hay should be added to stimulate gastrointestinal motility. In addition, cattle should be allowed to move freely. If considered by producers, these factors will decrease the occurrence of abdomen obstruction.

## Conclusion

This retrospective study showed that non-surgical therapy is the first choice of treatment for abdomen AI, with a cure rate of 61% (30/49), especially for cattle with mild and moderate cases. When non-surgical treatment is ineffective, surgical treatment can be selected. However, the treatment outcome in worsening cases is not ideal, and the cure rate in this study was only 23% (3/13), which is only suitable for those with moderate severity.

## Data Availability Statement

The original contributions presented in the study are included in the article/supplementary material, further inquiries can be directed to the corresponding author/s.

## Ethics Statement

The animal study was reviewed and approved by College of Veterinary Medicine, Gansu Agricultural University. Written informed consent was obtained from the owners for the participation of their animals in this study.

## Author Contributions

KY, ZL, QY, and HJ contributed to writing the manuscript and literature review. KY, ZL, CZ, and QZ contributed to critical revision of the manuscript as well as interpreting and describing the imaging findings. KY and ZL performed the described surgery on the patient. QZ and HJ assessed the gross and examined the biochemistry. YZ and SC contributed to critical revision of the manuscript, assisted with surgery on the patient, and managed the clinical case. All authors contributed to the final review.

## Conflict of Interest

The authors declare that the research was conducted in the absence of any commercial or financial relationships that could be construed as a potential conflict of interest.
